# Higher Desolvation Energy Reduces Molecular Recognition in Multi-Drug Resistant HIV-1 Protease

**DOI:** 10.3390/biology1010081

**Published:** 2012-05-31

**Authors:** Yong Wang, Tamaria G. Dewdney, Zhigang Liu, Samuel J. Reiter, Joseph S. Brunzelle, Iulia A. Kovari, Ladislau C. Kovari

**Affiliations:** 1 Department of Biochemistry and Molecular Biology, Wayne State University School of Medicine, Detroit, MI 48201, USA; Email: yowang@med.wayne.edu (Y.W.); tdewdne@med.wayne.edu (T.G.D.); zgliu0805@yahoo.com (Z.L.); sreiter@med.wayne.edu (S.J.R.); ikovari@med.wayne.edu (I.A.K.); 2 Department of Molecular Pharmacology and Biochemistry, Feinberg School of Medicine, Northwestern University, Chicago, IL 60611, USA; Email: j-brunzelle@northwestern.edu

**Keywords:** multi-drug resistant HIV-1 protease, x-ray crystallography, desolvation energy

## Abstract

Designing HIV-1 protease inhibitors that overcome drug-resistance is still a challenging task. In this study, four clinical isolates of multi-drug resistant HIV-1 proteases that exhibit resistance to all the US FDA-approved HIV-1 protease inhibitors and also reduce the substrate recognition ability were examined. A multi-drug resistant HIV-1 protease isolate, MDR 769, was co-crystallized with the p2/NC substrate and the mutated CA/p2 substrate, CA/p2 P1’F. Both substrates display different levels of molecular recognition by the wild-type and multi-drug resistant HIV-1 protease. From the crystal structures, only limited differences can be identified between the wild-type and multi-drug resistant protease. Therefore, a wild-type HIV-1 protease and four multi-drug resistant HIV-1 proteases in complex with the two peptides were modeled based on the crystal structures and examined during a 10 ns-molecular dynamics simulation. The simulation results reveal that the multi-drug resistant HIV-1 proteases require higher desolvation energy to form complexes with the peptides. This result suggests that the desolvation of the HIV-1 protease active site is an important step of protease-ligand complex formation as well as drug resistance. Therefore, desolvation energy could be considered as a parameter in the evaluation of future HIV-1 protease inhibitor candidates.

## 1. Introduction

Developing novel HIV-1 protease inhibitors is a current requirement to keep pace with the emergence of drug resistance mutations in the HIV-1 protease. Most of the current HIV-1 protease inhibitors are designed based on substrate mimicking. In addition to mimicking the transition state of the substrate cleavage, inhibitors designed based on the consensus volume of substrates have been developed [1]. The consensus volume of various substrates is defined as the substrate envelope. The HIV-1 protease substrate envelope has been determined based on the protease-substrate co-crystal structures [2]. A more realistic dynamic model of the substrate envelope has been refined using molecular dynamics of HIV-1 protease-substrate complex structures [3]. The study of the substrate binding to various HIV-1 protease variants, especially drug-resistant variants, facilitates the development of drugs inhibiting a broad panel of HIV-1 protease variants. The accumulated drug-resistance mutations in the protease may alter the subsites of the active site cavity, and therefore the inhibitor binding affinity alters accordingly. Studies have shown that the substrate analog inhibitors contact the same set of residues of HIV-1 protease as the natural substrates do [4]. However, substrates are more flexible than drugs and may compensate for the loss of some interactions [5]. Therefore, designing adaptive inhibitors to protease polymorphisms or drug-resistant variants may restore drug efficacy [6]. The first step of designing adaptive inhibitor is to study the formation of HIV-1 protease-substrate complexes with multi-drug resistant (MDR) HIV-1 proteases. 

Four clinical MDR HIV-1 protease variants, MDR 769, MDR 807, MDR 1385, and MDR 3761, were isolated by Palmer *et al*. [7], among which MDR 769 exhibited resistance to all the tested inhibitors [8] and was successfully crystallized. The accumulation of drug-resistance mutations also alters the structure of the MDR protease variants. The flaps of the apo MDR 769 protease adopt a wide-open conformation [9]. The diverse structures of apo HIV-1 protease variants or protease complexes have been imperative to drug design research. However, in the HIV protease structure database [10], only a few structures are of the wide-open form. Therefore, based on the available structures and known crystallization conditions, MDR 769 could serve as a model for studying the binding patterns of MDR protease variants.

Peptidomimetic design based on the HIV-1 protease substrates is one of the popular drug design strategies. In our study, four clinical isolates of MDR HIV-1 protease variants were examined. The four MDR HIV-1 protease variants exhibited resistance to all US FDA-approved HIV-1 protease inhibitors, and the substrate recognition ability is also reduced in these proteases. In the experiment of the HIV-1 protease substrate-based peptides competing with a fluorescent substrate, the substrate recognition of various HIV-1 protease variants was illustrated. The results showed different recognition of the substrates p2/NC, CA/p2 P1’F, RH/IN, and RT/RH among the five HIV-1 protease variants. With the co-crystal structures of p2/NC and CA/p2 P1’F with MDR 769 protease, five HIV-1 protease variants in complex with p2/NC and CA/p2 P1’F were modeled and simulated to study the binding of substrates in the protease active site cavity. The simulation results indicated that the MDR proteases need to overcome the higher desolvation energy barrier to form substrate-protease complexes.

## 2. Results and Discussion

### 2.1. The Four Clinical MDR HIV-1 Protease Isolates Are Resistant to All FDA-Approved HIV-1 Protease Inhibitors

The drug-resistance profiles of the four clinical MDR HIV-1 protease isolates were identified using enzyme inhibition assays. The four MDR protease variants are resistant to all of the FDA-approved HIV-1 protease inhibitors at various levels (Table 1). In the table, the HIV-1 protease variant NL4-3 represents a WT HIV-1 protease. Regarding to the inhibitory efficacy, the second generation of HIV-1 protease inhibitors (tipranavir, darunavir, lopinavir, and atazanavir) encountered lower relative resistance. Among the four MDR HIV-1 protease variants, MDR 807 and MDR 1385 were more resistant to the second generation HIV-1 protease inhibitors. For the first-line HIV-1 protease inhibitors [11], higher resistance was observed to atazanavir compared to darunavir. These results confirmed the in vitro resistance of the HIV-1 protease clinical isolates. 

**Table 1:** IC_50_ and fold resistance of multi-drug resistant HIV-1 protease variants.

HIV-1 Proteases	IC_50_ of HIV-1 protease inhibitors * in nM								
	DRV	ATV	LPV	TPV	NFV	APV	SQV	IDV	RTV
NL4-3	0.26	0.19	0.28	0.24	1.6	0.43	0.50	0.47	0.34
MDR 769	0.74	2.9	0.50	0.65	110	4.8	290	120	61
MDR 807	2.0	7.6	1.2	0.63	210	2.9	850	280	14.6
MDR 1385	3.0	4.6	2.3	2.8	230	3.1	29	140	8.0
MDR 3761	0.89	2.2	0.39	0.63	430	4.1	110	210	21

Inhibitor abbreviations: DRV (darunavir), ATV (atazanavir), LPV (lopinavir), TPV (tipranavir), NFV (nelfinavir), APV (amprenavir), SQV (saquinavir), IDV (indinavir), RTV (ritonavir). * The protease inhibitors were requested from the NIH AIDS Research and Reference Reagent Program (www.aidsreagent.org).

### 2.2. The MDR HIV-1 Protease Isolates Exhibited Different Substrate Binding Preference Relative to the WT Protease

**Figure 1:**
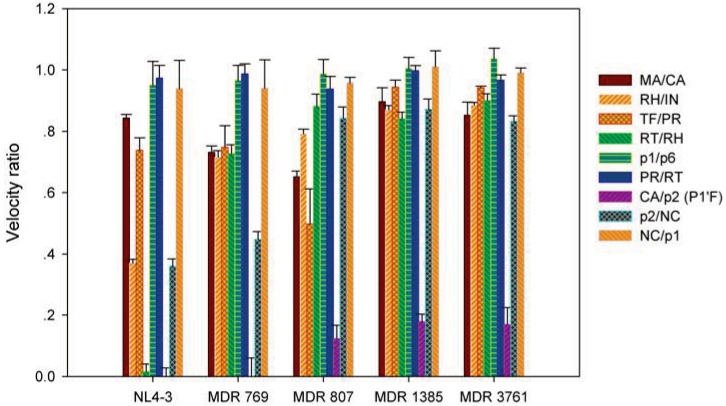
Förster resonance energy transfer substrate processing ratio. The bar chart represents the ratio of the average FRET substrate cleavage velocity in the presence of the regular peptide substrate over the average FRET substrate cleavage velocity in the absence of the regular peptide substrate. When the velocity ratio is one, the regular peptide does not affect the FRET substrate cleavage by the protease. When the velocity ratio is zero, the FRET substrate is completely competed out by the regular peptide.

Compared to inhibitors, the higher flexibility of substrates could match the dynamic changes in the protease [12]. The study of substrate binding facilitates the development of peptidomimetic inhibitors. In the FRET substrate cleavage interference experiments, the five HIV-1 protease variants show varied preferences to the nine heptapeptides. The processing ratio in Figure 1 represents the ratio of the average FRET substrate processing velocity in the presence of the heptapeptide over the average FRET substrate processing velocity in the absence of the heptapeptide. The higher ratio indicated less interference by the peptide on FRET substrate processing. Both the peptide and the FRET substrate were in excess molar ratio to the HIV-1 protease, and the peptide concentration was 40-fold higher than the FRET substrate concentration. The rate-limiting step of the HIV-1 protease substrate, catalysis, is a chemical process rather than a physical process [13]. Therefore, interference was caused by the peptide cleavage process rather than the peptide binding process. Once a protease-substrate complex is formed, the cleavage of FRET substrate is slowed down. The high velocity ratios of the FRET substrate cleavage with or without the regular peptide were due to the lower chance of forming protease-substrate complex with the peptide. The complex formation theory was supported by the enzyme assays using the uncleavable CA/p2 P1’F peptide. The inhibition of the uncleavable peptide is correlated with the peptide competition results. The IC_50_ values of CA/p2 P1’F pseudopeptide for NL4-3, MDR 769, MDR 807, MDR 1385, and MDR 3761 were 2.6 nm, 4.4 nm, 16.0 nm, 32.5 nm, and 55.4 nm, respectively. The corresponding relative resistance values were 1.7, 6.2, 13, and 21 fold, respectively. The correlation coefficient between the FRET velocity ratios in presence of CA/p2 P1’F and the IC_50_ values of CA/p2 P1’F pseudopeptide was 0.86.

The ratios of heptapeptide interference exhibited the likelihood of protease-substrate complex formation (Figure 1). Compared to the WT protease, the MDR protease variants were interfered at lower levels by the presence of regular peptides indicating the impaired ability of the MDR HIV-1 protease to form a complex with the substrates. All five HIV-1 protease variants had higher probability of forming complexes with the CA/p2 P1’F peptide. The presence of CA/p2 P1’F significantly slowed down the processing of the FRET substrate. Especially, the NL4-3 and MDR 769 were completely occupied by CA/p2 P1’F, and the fluorescent signal maintained as the base line of background signal. The MDR 807, MDR 1385, and MDR 3761 had higher residual velocities of FRET substrate processing with the interference of CA/p2 P1’F, suggesting the decreased probability of complex formation. The MDR 807, MDR 1385, and MDR 3761 exhibited a lower probability of forming complex with the substrate p2/NC compared to the WT protease and MDR 769. All four MDR protease variants were unfavorable to form complex with the RT/RH and RH/IN.

During the viral maturation, the processing of Gag and Gag-pol polyprotein is an ordered process [14]. The WT HIV-1 protease should maintain varying binding affinities to the different substrates. However, in the substrate interference assays, the FRET substrate cleavage interference pattern of the WT HIV-1 protease is not preserved in the assays of MDR proteases. MDR proteases acquire drug-resistance by decreasing the probability of binding to inhibitors. It is also possible for the mutations and polymorphic substitutions to decrease the ability of MDR HIV-1 protease to bind substrates. The change in regulated substrate site processing could be a mechanism to compensate for the loss of viral fitness and could be a mechanism to diverse the MDR HIV-1 variant quasispecies populations in an infected individual. However, the short substrate peptides used in this study may not reflect the processing order of the polyproteins cleavage sites because the structural context and protease accessibility were not evaluated. 

### 2.3. The MDR Protease-Substrate Co-Crystal Structures Were Insufficient to Explain the Different Substrate Binding Behaviors between the MDR and WT HIV-1 Proteases

**Figure 2:**
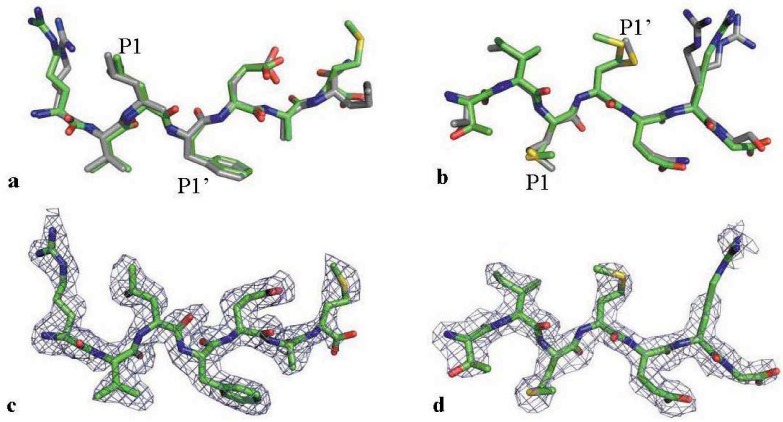
Substrate conformation illustrating binding to the MDR protease. (**a**) The CA/p2 P1’F binding to the MDR 769 (green) comparing to its binding to a WT protease (grey, PDB ID: 1A8K); (**b**) The p2/NC binding to the MDR 769 (green) comparing to its binding to a WT protease (grey, PDB ID: 1KJ7); (**c**) The electron density of CA/p2 P1’F. The mesh is an *Fo-Fc* OMIT map at 2.0 σ; (**d**) The electron density of p2/NC. The mesh is an *Fo-Fc* OMIT map at 2.0 σ.

Based on substrate competition experiments, the substrates of interest were selected to co-crystallize with the MDR HIV-1 protease variant. The peptides CA/p2 P1’F and p2/NC were successfully crystallized with MDR 769 in *P2_1_2_1_2_1_* space group. The CA/p2 P1’F-MDR 769 and p2/NC-MDR 769 co-crystals diffracted to 2.10 Å and 2.30 Å, respectively (Table 2). The two structures were deposited to the Protein Data Bank. The access codes for CA/p2 P1’F-MDR 769 and p2/NC-MDR 769 co-crystal structures are 4FAF and 4FAE, respectively. MDR 769 adopts wide-open flap conformation in the absence of ligand [9]. However, the peptide binding results in closing of the MDR 769 protease flaps as is the case for the wild-type protease complex. By superposing the MDR-substrate complex structures on corresponding WT protease-substrate complexes, the substrate backbones were overlapped in both the MDR and WT complexes, and only minor conformational differences were noticeable in the long flexible side chains (Figure 2a,b). The P1’ group of p2/NC in complex with MDR 769 was the major conformational deviation as compared to that of the WT complex. The results suggested that the static structures of binding conformation are insufficient to explain the difference in substrate recognition of HIV-1 protease variants. Dynamic simulations are required to identify the sophisticated differences in substrate binding among HIV-1 protease variants.

### 2.4. The Desolvation Energy Required by the MDR HIV-1 Protease Variants to Form Protease-Substrate Complexes Correlated with the Substrate Binding Assay

**Figure 3:**
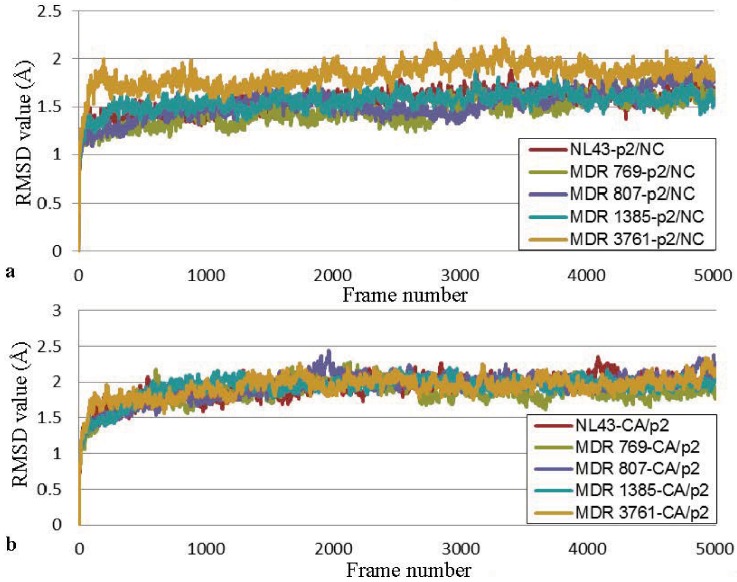
RMSD values of the HIV-1 protease-peptide complexes. (**a**) RMSD values of the HIV-1 protease-p2/NC complexes; (**b**) RMSD values of the HIV-1 protease-CA/p2 complexes.

Homology complex models of MDR 769, MDR 807, MDR 1385, MDR 3761, and NL4-3 with p2/NC or CA/p2 were built to analyze the dynamic interactions between substrate and protease. After 10 ns molecular dynamics simulation, the HIV-1 protease complex models became relatively stable (Figure 3), and the movement of substrates was analyzed for the last 40 ps of simulation.

The electrostatic desolvation energy is an unfavorable contribution to the binding free energy of HIV-1 protease-substrate complexes. Compared to the electrostatic desolvation energy, the non-polar desolvation energy was not a large component for the binding of HIV-1 protease to p2/NC or CA/p2. The desolvation energy changes for MDR proteases in complex with CA/p2 were higher than that for the WT complex. The p2/NC complexes with MDR 807, MDR 1385, and MDR 3761 were calculated to have higher desolvation energy than MDR 769 and NL4-3 complexes. Generally, MDR proteases required to overcome higher desolvation barriers to bind substrates.

**Table 2:** Crystallographic statistics.

Dataset		The MDR 769 in complex of substrate CA/p2	The MDR 769 in complex of substrate p2/NC
**Data collection**			
Space group		*P2_1_2_1_2_1_*	*P2_1_2_1_2_1_*
Wavelength (Å)		0.979	0.979
Cell constants (Å)		a = 28.76 b = 65.38 c = 92.80	a = 28.62 b = 63.85 c = 91.11
Resolution range (Å)		30.00−2.10 (2.14−2.10)	30.00−2.30 (2.38−2.30)
Number of unique reflections		10882 (507)	7945 (787)
Completeness (%)		99.9 (99.0)	98.8 (99.5)
Redundancy		7.6 (6.0)	4.0 (4.0)
Mean I/σ (*I*)		13.2 (3.4)	10.0 (2.4)
*R_merge_* ^a^		0.162 (0.520)	0.114 (0.451)
**Refinement**			
*R_work_* (%)^b^		17.29	20.00
*R_free_* (%)^b^		23.99	27.86
Number of atoms			
	Ligand	60	56
	Protease	1529	1529
	Solvent	258	137
Average isotropic B factor (Å^2^)			
	Ligand	20.28	35.32
	Protease	16.83	30.46
	Solvent	32.62	45.32
RMSD bond length (Å)		0.008	0.009
RMSD bond angle (°)		1.06	1.26
Ramachandran plot			
Allowed/generous/disallowed (%)		100/0/0	99.0/1.0/0

*^a^ R_merge_* *= Σ*_hkl_* Σ*_i_* |*I_i_ (hkl) *− <*I(hkl)*>|* */* *Σ*_hkl_ *Σ*_i_ I_i_(hkl), where I_i_ (hkl) is the intensity of an observation and I(hkl) is the mean value for its unique reflection. *^b^ R_work_* =*Σ*_hkl_ ||F_o_|−|F_c_|| / *Σ*_hkl_ |F_o_|, where F_o_ and F_c_ are the observed and calculated structure factor amplitudes. *R_free_* is calculated exactly as *R_work_* using a random 5% of the reflections omitted from refinement.

The energy barrier of desolvation correlated well with velocity ratios in substrate competition experiments. The desolvation energy (ΔG_desolv_) includes the non-polar desolvation energy (ΔG_desolv_^nonpolar^) and the electrostatic desolvation energy (ΔG_desolv_^elec^). The MDR proteases require higher desolvation energy to remove the water shell in the active site to form complexes with the substrate (Table 3, Table 4). Pearson’s correlation coefficient between the velocity ratios for the p2/NC peptide (Figure 1) and the desolvation energies of the p2/NC-MDR protease complexes is 0.87. Pearson’s correlation coefficient for the CA/p2 dataset is 0.91.

The desolvation energy is an important component for substrate and inhibitor recognition. The higher unfavorable desolvation energies of CA/p2-MDR complexes explain the resistance to the uncleavable CA/p2 reduced peptide. In the dynamic process of ligand binding, desolvation energy plays an important role in the ligand entry and protease-ligand complex formation. The MDR proteases required a higher energy to desolvate the substrate and the protease active site. The higher desolvation barrier for MDR proteases increases the difficulty of protease-ligand complex formation. The input structures for simulation were modeled based on the closed form MDR 769. Both the crystal structure and direct measurement using pulsed double electron-electron resonance (DEER) have confirmed a larger distance between the flaps of MDR 769 as compared to the wild-type protease structure [15,16]. The wide-open conformation of the MDR protease in the solution exposes more active site regions to solvent which may further elevate the desolvation barrier. Since the calculation of interaction energy and desolvation energy were performed using different methods, it is not appropriate to compare directly between the two datasets. However, the trends of energy difference among HIV-1 protease complexes are good indicators for interpretation.

**Table 3:** Energy analysis of the HIV-1 protease-p2/NC complex.

Terms of binding free energy (kcal/mol)	HIV-1 protease				
	NL4-3	MDR 769	MDR 807	MDR 1385	MDR 3761
ΔG_desolv_^elec^	178 ± 10	188 ± 11	210 ± 16	228 ± 19	256 ± 10
ΔG_desolv_^nonpolar^	−7.6 ± 0.2	−7.7 ± 0.2	−7.9 ± 0.2	−7.6 ± 0.2	−7.7 ± 0.1
ΔG_desolv_	170	180	202	220	248

**Table 4:** Energy analysis of the HIV-1 protease-CA/p2 complex.

Terms of binding free energy (kcal/mol)	HIV-1 protease				
	NL4-3	MDR 769	MDR 807	MDR 1385	MDR 3761
ΔG_desolv_^elec^	278 ± 22	283 ± 25	315 ± 18	302 ± 20	330 ± 18
ΔG_desolv_^nonpolar^	−7.5 ± 0.1	−7.8 ± 0.2	−7.6 ± 0.2	−6.9 ± 0.1	−7.3 ± 0.1
ΔG_desolv_	271	275.	307	295	322

## 3. Experimental Section

### 3.1. Protein Expression and Purification

Table 5 lists the protein sequences of MDR HIV-1 proteases. Active and inactive MDR HIV-1 protease genes were codon optimized for *E. coli* expression, synthesized by GENEART, Inc. (Regensburg, Germany), and inserted into the pET21b plasmid. To prevent auto-proteolyses, Q7K mutation was introduced into the active MDR genes. The A82T mutation was introduced to facilitate crystallization. The protein expression, purification, and refolding procedures were described previously [15]. The proteases prepared for crystallization were concentrated to 1.5 mg/mL in the buffer of 20 mM sodium acetate, pH 5.0, 1 mM dithiothreitol (DTT), and 10% (v/v) glycerol using Amicon concentrators with 5 kDa molecular mass cut-off (Millipore Corporate, Billerica, MA, USA).

### 3.2. Protease Inhibition and Substrate Interference Assays

HIV protease Forster Resonance Energy Transfer (FRET) substrate I used in the IC_50_ determination and substrate interference was purchased from AnaSpec, Inc. (Fremont, CA, USA). The US FDA-approved HIV-1 protease inhibitors were kindly provided by the NIH AIDS Research and Reference Reagent Program (www.aidsreagent.org). The CA/p2 pseudopeptide with a reduced scissile peptide bond [-ψ(CH2NH)-] was synthesized in the Department of Chemistry, Wayne State University. The nine substrate hepta-peptides were purchase from SynBioSci Corporation, Livermore, CA, USA. (Table 6). All the hepta-peptides were purified by HPLC to purity higher than 98%. The IC_50_ determination was described previously [8]. The procedure of substrate interference assay was similar to the protease inhibition assay except that inhibitor was replaced by regular substrate. The HIV-1 protease reaction buffer was 0.1 M sodium acetate, 1.0 M sodium chloride, 1.0 mM ethylenediaminetetraacetic acid (EDTA), 1.0 mM DTT, 10% dimethylsulfoxide (DMSO), and 1 mg/mL bovine serum albumin (BSA) at pH 4.7. The protease concentration was adjusted to a substrate cleavage velocity of 5 Relative Fluorescence Units (RFU)/min in the absence of regular substrate. In the enzyme reaction buffer, regular HIV-1 protease substrates, heptapeptides, was mixed with the FRET HIV-1 protease substrate to reach a final concentration of 100 μM and 2.5 μM, respectively. Upon the addition of the HIV-1 protease, the fluorescent signal emitted through the cleavage of the FRET substrate was recorded using SpectraMax M5 (Molecular Devices, Sunnyvale, CA, USA) at an excitation wavelength of 340 nm and an emission wavelength of 490 nm. The average velocity of FRET substrate cleavage in RFU/min was calculated based on fluorescent signal at 30 time point over 30 min. In the control experiment, the average velocity of FRET substrate cleavage was determined in the absence of regular substrates. The result was illustrated by bar chart representing the ratio of the average FRET substrate cleavage velocity in the presence of regular substrates over the average FRET substrate cleavage velocity in the absence of regular substrates.

**Table 5:** Sequences of HIV-1 protease variants.

Residues	HIV-1 protease	Sequences *
1–50	NL4-3	PQITLWKRPL VTIKIGGQLK EALLDTGADD TVLEEMNLPG RWKPKMIGGI
	MDR 769	PQITLWKRPI VTIKIGGQLK EALLDTGADD TVLEEVNLPG RWKPK**L**IGGI
	MDR 807	PQITLWKRPI VTIKIGGQLK EALLDTGADD TVLEEMNLPG KWKPK**I**IVGI
	MDR 1385	PQITLWKRP**F** VTIKIGGQLK EALLDTGADD TVLEEIDLPG RWKPK**I**IGGI
	MDR 3761	PQITLWKRPI VAIKVGGQII EALLDTGADD TVLEEMNLPG RWKPK**I**IGGI
51–99	NL4-3	GGFIKVRQYD QILIEICGHK AIGTVLVGPT PVNIIGRNLL TQIGCTLNF
	MDR 769	GGF**V**KVRQYD QVPIEICGHK VIGTVLVGPT P**A**N**V**IGRNL**M** TQIGCTLNF
	MDR 807	GGF**T**KVRQYD NVQIEICGHK VIG**A**VLIGPT P**A**NIIGRNLL TQLGCTLNF
	MDR 1385	GGFIKVKQYD QIPIEICGHK VIGTVLVGPT P**T**NIIGRNM**M** TQLGCTLNF
	MDR 3761	GGFIKVRQYD QIP**V**EICGHK **I**I**T**TVLVGST PVN**V**IGRNL**M** TQLGCTLNF

* The polymorphic changes are underlined. The drug-resistance mutations are in bold.

### 3.3. Crystallization, Data Collection, and Structure Refinement

The substrates (p2/NC and CA/p2 P1’F) were co-crystallized with the MDR769 inactive protease by the hanging drop vapor diffusion method. The protease-substrate mixture (molar ratio 1:20) was then mixed at 2:1 v/v ratio with the mother liquor. The MDR 769-p2/NC complex was crystallized in 0.1 M citric acid and 2.4 M ammonium sulfate at pH 5.2 while the MDR 769-CA/p2 complex was crystallized in 0.1 M MES and 2.4 M ammonium sulfate at pH 6.0. The reservoir volume was 750 µL. Needle shape crystals grew to a suitable size for diffraction within a week. The crystals were cryoprotected with 30% (w/v) glucose in the reservoir solution before being frozen in liquid nitrogen. Diffraction data were collected at the Life Sciences Collaborative Access Team (LS-CAT) at the Advanced Photon Source (APS) Sector 21, Argonne National Laboratory (Argonne, IL, USA) and processed with HKL2000 program suite [17]. The structures were solved by molecular replacement using a previously solved MDR 769 complex structure (PDB: 3SPK) as a searching model and refined using REFMAC5 of CCP4 suite [18]. 

**Table 6:** Sequences of the nine HIV-1 protease cleavage sites within the HIV-1 Gag-Pol polyprotein.

substrate	P3	P2	P1	P1’	P2’	P3’	P4’
MA/CA	Gln	Asn	Tyr	Pro	Ile	Val	Gln
CA/p2*	Arg	Val	Leu	Phe	Glu	Ala	Met
p2/NC	Thr	Ile	Met	Met	Gln	Arg	Gly
NC/p1	Gln	Ala	Asn	Phe	Leu	Gly	Lys
p1/p6	Gly	Asn	Phe	Leu	Gln	Ser	Arg
TF/PR	Phe	Asn	Phe	Pro	Gln	Ile	Thr
PR/RT	Leu	Asn	Phe	Pro	Ile	Ser	Pro
RT/RH	Glu	Thr	Phe	Tyr	Val	Asp	Gly
RH/IN	Lys	Ile	Leu	Phe	Leu	Asp	Gly

The cleavage site is between P1 and P1’ residue. The CA/p2 was introduced with an alanine to phenylalanine mutation at P1’ position in order to increase its binding affinity to HIV-1 protease.

### 3.4. Molecular Dynamics Simulation

The X-ray structures of the protease-substrate complex (MDR 769 in complex with the substrate p2/NC) is solved and used as an initial structure for homology modeling. The complexes of MDR 807-p2/NC, MDR 1385-p2/NC, MDR 3761-p2/NC, and NL43-p2/NC were constructed using SWISS-MODEL [19]. Similarly, a series of HIV-1 protease-CA/p2 complexes were built based on the MDR 769-CA/p2 co-crystal structure. Based on the catalytic mechanism of HIV-1 protease, Asp 25 was assigned as a protonated state while Asp 25’ was assigned as a deprotonated state. All histidine residues were assigned a neutral charge. Protonation states of other amino acid residues were assumed based on the buffer pH in the HIV-1 protease enzymatic assays (pH 4.7). To avoid simulating a catalytic interaction, positional restraints were applied to the scissile peptide bond of the substrate and the β-carboxyl groups of catalytic residue Asp 25 and Asp 25’. The HIV protease-substrate complex was placed into an orthogonal TIP3P water box. The TIP3P is a three-site (three interaction sites) water model, which is one of the most widely used water models. The protease complex was at least 12 Å from the edge of the water box. Na^+^ and Cl^−^ ions were added to neutralize the simulation system.

The MD simulations were performed using the parallel computing program Scaling Nano Molecular Dynamics (NAMD) V. 2.7b [20]. The protease complex models were solvated in a water box using TIP3P models for water molecules. To prevent translational and rotational displacement and to prevent the simulation of the catalytic reaction, positional restraints were applied for the carbonyl group of the substrate scissile peptide bond and the catalytic residue Asp 25 and Asp 25’ of the HIV-1 protease. The cutoff for non-bonded interactions was 10 Å. Particle Mesh Ewald was implemented; the Particle Mesh Ewald method calculates direct-space interactions within a finite distance using a modification of Coulomb’s law, and in reciprocal space using a Fourier transform to build a “mesh” of charges, interpolated onto a grid [21]. The systems were energy minimized using a conjugate gradient method and gradually heated from 70K to 310K in 200 ps. Simulations were conducted at 310K and 1.0 atm (NPT ensemble) for 10 ns using the CHARMM force field 27 and a timestep of 2 fs. 

### 3.5. Energy Calculations

Trajectories of MD simulation were visualized and analyzed using the Visual Molecular Dynamics (VMD) program V. 1.91. The superposition of molecular structures was carried out using Pymol. The root-mean-square deviation (RMSD) values were calculated using the VMD RMSD trajectory plug-in. Previous studies demonstrated that HIV-1 protease ligands show single-maxima probability density function of energy [22]. Therefore, the last 500 ps simulation represents a relatively stable protease-ligand complex conformation. Hydrophobic solvation energy and electrostatic solvation energy were calculated and averaged based on the 100 snapshot coordinates of the last 500 ps simulation. 

The change in desolvation energy during the binding of protease to its substrate was obtained with the following equation, ΔG_solv_ = ΔG_desolv_^electrostatic^ + ΔG_desolv_^nonpolar^. The electrostatic desolvation energy was calculated using the Adaptive Poisson-Boltzmann Solver (APBS) program [23], a software package for modeling biomolecular solvation through solution of the Poisson-Boltzmann equation (PBE). The electrostatic desolvation energy upon protease-substrate complex formation was calculated using the following equation, ΔG_desolv_^elec^ = ΔΔG_solv_^elec^ = ΔG_solv_^elec,complex^ – ΔG_solv_^elec,protease^ – ΔG_solv_^elec,substrate^. The symbol ΔG_solv_ refers to the difference of solvation energy when a molecule is transferred from water solution (with a dielectric constant of 78.54) to vacuum (with a dielectric constant of 1.00). The symbol ΔΔG_solv_^elec^ represents the solvation energy change (desolvation energy) upon protease-substrate complex formation. The input for the APBS program was prepared by converting the PDB format coordinate to PQR format through the PDB2PQR server (http://propka.ki.ku.dk), an automated pipeline for the setup, execution, and analysis of Poisson-Boltzmann electrostatics calculations [24]. The PDB2PQR server adds charge and radius parameters to existing PDB data. The non-polar desolvation energy was calculated using the equation ΔG_desolv_^nonpolar^ = – ΔG_solv_^nonpolar^ = – (γΔSASA + β), where SASA stands for solvent accessible surface area, γ is a standard value of 0.00542 kcal*mol^−1^*Å^−2^, and β is 0.92 kcal mol^−1^ [25]. The change of solvent accessible surface area upon protease-substrate complex formation is calculated using the following equation, ΔSASA = SASA^complex^ − SASA^protease^ − SASA^substrate^.

## 4. Conclusions

The study of MDR HIV-1 protease substrate preference in binding and catalysis improves the understanding of MDR HIV-1 protease evolution as well as the effective inhibition of the MDR protease variants. Novel drugs could be designed or optimized according to the favorable binding of substrates to the MDR HIV-1 protease.

Based on the enzyme assays and simulation results, we can conclude that the difficulty in protease-ligand complex formation is increased for MDR protease due to the high desolvation energy barrier. Drug design purely based on the enzyme of inhibited state may not be able to reproduce the dynamic interactions during the formation of enzyme-inhibitor complex. The results in this chapter suggest that the desolvation of the HIV-1 protease active site is an important step in protease-ligand complex formation as well as drug resistance. Since it is computationally costly to simulate the process of inhibitor-enzyme recognition, desolvation energy could serve as a simplified parameter to be considered in drug design. The evaluation of desolvation energy could be utilized as one standard to screen drug candidates.
